# Assessing the Identification and Treatment of Post-Stroke Depression: An Audit in a Stroke Rehabilitation Ward

**DOI:** 10.1192/j.eurpsy.2026.11710

**Published:** 2026-07-31

**Authors:** Z. Yaqoob, S. Ponnambath, O. Balogan

**Affiliations:** 1 School of Medicine, Cardiff University, Cardiff; 2 Stroke, Grange University Hospital, Cwmbran, United Kingdom

## Abstract

**Introduction:**

Post-stroke depression (PSD) is a common neuropsychiatric complication, affecting around one-third of stroke survivors. It is linked to poorer functional recovery, higher mortality, and reduced rehabilitation participation. NICE stroke guidelines recommend routine mood screening within 6 weeks using validated tools, with timely referral to psychological or psychiatric services as needed. Despite this, practice is often inconsistent, leaving many patients undetected or untreated. This audit evaluated whether patients admitted to the stroke rehabilitation ward in Aneurin Bevan University Health Board (ABUHB) were screened and managed for PSD according to NICE guidance.

**Objectives:**

This audit aimed to evaluate adherence to NICE guidance on psychological assessment and management of post-stroke depression in patients admitted to the stroke rehabilitation ward in Caerphilly. Objectives were:

- **Mood Screening:** Determine whether all stroke patients were screened for mood disturbance within 6 weeks.
- **Follow-up Screen:** Assess whether patients received repeat screening at discharge or 6 months.
- **Identification of Depression:** Establish the proportion identified with post-stroke depression.
- **Treatment and Referral:** Determine whether patients with depression were offered appropriate intervention (psychological therapy, antidepressants, or referral).
- **Mental Health Involvement:** Review the involvement of clinical psychologists or other mental health professionals in care.

**Methods:**

A retrospective case-note audit was conducted on patients admitted to the stroke rehabilitation ward in Caerphilly. All patients with a confirmed stroke diagnosis (ischaemic or haemorrhagic) were included. Patients without stroke, discharged before mood screening, or with insufficient records were excluded. Data were collected from electronic and paper notes, MDT entries, and psychology referrals. Each patient received a unique anonymised identifier. Extracted data included demographics (age, gender, date and type of stroke), mood screening within 6 weeks, screening tools used, follow-up mood assessment (discharge or 6 months), documentation of post-stroke depression, treatment or referrals offered (psychological therapy, antidepressants, clinical psychology), involvement of mental health professionals, and relevant clinical notes (e.g., barriers such as communication or cognitive impairment). Audit standards were based on NICE Stroke Guidelines (CG162) and Stroke Quality Standard (QS2): 100% should receive mood screening within 6 weeks; should have follow-up at discharge or 6–12 months; all identified with depression should be offered treatment or referral. Data were collated in Excel then analysed for compliance.

**Results:**

Data collection is ongoing. Preliminary results will be available and presented at the conference.

**Conclusions:**

Data collection is ongoing. Preliminary results will be available and presented at the conference.

**Disclosure of Interest:**

None Declared

